# The mRNA expression and enzymatic activity of three enzymes during embryonic development of the hard tick *Haemaphysalis longicornis*

**DOI:** 10.1186/s13071-019-3360-8

**Published:** 2019-03-12

**Authors:** Tian-Tian Zhang, Zhao-Xi Qiu, Yuan Li, Wen-Ying Wang, Meng-Meng Li, Pei Guo, Jing-Ze Liu

**Affiliations:** Hebei Key Laboratory of Animal Physiology, Biochemistry and Molecular Biology, College of Life Sciences, Hebei Normal University, Shijiazhuang, 050024 China

**Keywords:** *Haemaphysalis longicornis*, Cathepsin B, Cathepsin D, Acid phosphatase, Embryonic development

## Abstract

**Background:**

Three main enzymes including cathepsin B, cathepsin D and acid phosphatase are involved in vitellin degradation, which is a major biochemical event of the embryonic development and can provide nutrients and metabolites for tick embryos. In the present study, the mRNA expression profiles and enzymatic activity of cathepsin B, cathepsin D and acid phosphatase were investigated during embryonic development in the tick *Haemaphysalis longicornis*.

**Results:**

The results revealed that all three enzymes were expressed throughout embryonic development. Both cathepsin B and acid phosphatase transcripts were accumulated during the first four days. Cathepsin B reached its highest expression on day 5, whereas the peak expression of acid phosphatase and cathepsin D occurred on day 11. The highest activity of cathepsin B was observed on the first day of egg development, whereas cathepsin D reached its highest activity on day 13. Acid phosphatase activity increased gradually during the first five days and then remained stable until the end of egg development.

**Conclusions:**

Three enzymes were expressed and activated in eggs, and also presented different dynamic changes with the development of embryos. The profiles of both mRNA expression and enzymatic activity of these enzymes indicate that they are controlled orderly and play multiple roles during embryonic development in ticks.

## Background

Ticks are obligatory hematophagous ectoparasites of wildlife, domestic animals and humans. They are notorious vectors that can transmit various pathogens (viruses, rickettsiae, bacteria, spirochaetes and protozoans) among arthropods [1, 2]. With climate change, new tick-borne pathogens and tick-borne diseases have become severe threats to public health all over the world [3, 4]. With a high reproductive potential, the fully engorged female can lay up to several thousand eggs [5]. After engorgement, the body weight of female ticks is increased nearly 100-fold compared to unfed ticks, and more than 50% of engorgement weight is transformed into eggs [6]. Therefore, one of the strategies to control tick-borne diseases is to explore new molecular targets for the interruption of the tick life-cycle. Embryonic development in eggs, the only non-parasitic stage, is vital in the tick life-cycle.

In ticks, vitellin (Vn) is a phosphorylated heme-lipoglycoprotein and its degraded products provide the source of amino acids, carbohydrates, heme, and other nutrients for embryonic development and unfed larva [7]. Regulatory mechanisms for the utilization of Vn have been implicated to rely on a variety of proteolytic enzymes in different species of insects [8–10]. Among them, three enzymes including cathepsin B [9, 11, 12], cathepsin D [13–15] and acid phosphatase [14, 16] have been extensively investigated in insects. Cathepsin B is a cysteine protease involved in a wide range of biological processes, including the degradation of regulatory proteins and digestive processes [8, 17]. As a proteolytic enzyme, cathepsin B has been reported to play an essential role in the embryonic degradation of Vn in insects [8, 9]. Cathepsin D is an aspartic protease mainly found in lysosomes. It is an intracellular digestive enzyme and is involved in the degradation or activation of proteins, hormones and other substrates [14, 16]. During the embryonic development of the triatomine *Rhodnius prolixus*, cathepsin D is an essential regulator for yolk protein degradation [16]. Acid phosphatase as an enzyme in the lysosome can catalyze the hydrolysis of orthophosphoric monoester from a wide variety of substrates [18]. It has been reported that acid phosphatase and cysteine protease cooperate to assure Vn breakdown during early embryogenesis of *Periplaneta americana* [19]; this is because acid phosphatase is able to dephosphorylate Vn, which increases its degradation by other proteolytic enzymes.

Compared to insects, the functional roles of three enzymes in Vn degradation in ticks and the regulatory mechanisms are still poorly understood. Previous studies which were only based on one species of tick, *Rhipicephalus* (*Boophilus*) *microplus*, have suggested that some enzymes may play different roles in Vn degradation *in vitro* [20–23]. However, the dynamic changes of these three enzymes during embryonic development are unknown. Meanwhile, knowledge on stage-specific proteolysis of Vn during tick embryo development and the relationship between different enzymes during this process remains scarce.

The hard tick *Haemaphysalis longicornis* is widely distributed in Australia, New Zealand, Korea, Japan and 17 provinces of China [24]. Moreover, it can transmit a large variety of pathogenic microorganisms to humans and animals, including *Theileria* spp., *Babesia gibsoni*, *Rickettsia* and *Coxiella burnetti* [25]. Recently, it was found for the first time that a large number of *H. longicornis* infested sheep in Hunterdon County, New Jersey, USA, suggesting an invasion of *H. longicornis* which presents a significant threat to human and animal health in the USA [26]. In our previous work, we reported the vitellogenesis in *H. longicornis* and its hormone regulation during ovary development [27]. Furthermore, we purified and characterized the properties of Vn from *H. longicornis*, finding that Vn from *H. longicornis* was composed of eight subunits with molecular weights of 112, 103, 80, 78, 71, 68, 62 and 52 kDa, respectively [28].

In the present study, we investigated the mRNA expression profiles and enzymatic activity of cathepsin B, D and acid phosphatase during embryonic development in *H. longicornis*, aiming to reveal the existence and dynamic changes of three enzymes during embryonic development in tick *H. longicornis*.

## Methods

### Sample collection and rearing

Free-living *H. longicornis* ticks were collected on vegetation in Xiaowutai National Natural Reserve Area in China by flag dragging and reared on domestic rabbits, *Oryctolagus cuniculus*, as described by Liu et al. [29]. The rabbits were maintained at 20–25 °C with 50% RH and exposed to natural daylight cycles, and each rabbit was used only for a single infestation. After engorgement, ticks were collected and incubated for oviposition in an incubator with 75 ± 5% relative humidity (RH) and 8:16 h of a light-dark cycle (L:D) at 26 ± 1 °C. A group of eggs were collected to obtain samples during oviposition every day at the same time and kept separately in the incubator, then frozen quickly using liquid nitrogen when they reached different developmental stages and stored at -80 °C for further analysis. A control group of eggs was allowed to develop completely to determine the exact day of hatching.

### RNA extraction and cDNA synthesis

Total RNA from each developmental stage (100 mg of eggs) was extracted using the AxyPrep™ Multisource Total RNA Miniprep Kit (Axygen, San Jose, USA) following the manufacturer’s protocol. The RNA concentration and purity were determined using a Nano Drop^®^ ND-1000 Spectrophotometer (Thermo Fisher Scientific, Waltham, MA, USA) and RNA integrity was evaluated by agarose gel electrophoresis. RNA samples were stored at -80 °C until use. The complementary DNA (cDNA) was synthesized from 4 μg of total RNA by reverse transcription reaction using TransScript^®^ One-Step gDNA Removal and cDNA Synthesis SuperMix (TransGen Biotech, Beijing, China), which excluded genomic DNA contamination. The cDNA samples were stored at -20 °C until use.

### Primer design

Primers were designed using the information from the GenBank sequences for different kinds of enzymes in *H. longicornis*. The genes chosen for this study are presented in Table 1. For cathepsin B, cathepsin D and acid phosphatase, primers were designed using the cathepsin B sequence (AN AB255051), aspartic protease sequence (AN EU019715) and lysosomal acid phosphatase sequence (AN HM150759) from *H. longicornis*, respectively. For control actin gene, primers were designed using the β-actin sequence (AN AY254898) from *H. longicornis*. All primers were designed with Primer Premier 5 considering a theoretical optimal annealing temperature of about 60 °C, with a melting temperature (Tm) difference between the forward and reverse primers not greater than 5 °C, guanine and cytosine percentages between 40–60%, primer lengths of 18–22 bp and free of dimers and hairpins.

**Table 1:** Primers for genes evaluated in the present study

Gene	Primer sequence (5′-3′)	Tm (°C)	Amplicon size (bp)
Cathepsin B	F: CAACTCCTGGAACACCGAA	59.7	78
	R: GTCTTCAATGCCGCACTCA	61.0	
Cathepsin D	F: CTCGGTTCTGAATGTGCCA	60.1	165
	R: GGTGATGTTGCCAGTGTAGTG	59.7	
Acid phosphatase	F: GAGACAGATGCCTGGAGAGC	60.8	71
	R: TCCACACTTTCTTGTCCCG	59.1	
β-actin	F: CGTTCCTGGGTATGGAATCG	62.5	70
	R: TCCACGTCGCACTTCATGAT	62.7	

*Abbreviation*: Tm, melting temperature

### Amplicon sequence analysis

Positive polymerase chain reaction (PCR) products were purified and cloned into pEASY^®^-T1 Simple Cloning Vector (TransGen Biotech, Beijing, China) using pEASY^®^-T1 Simple Cloning Kit (TransGen Biotech, Beijing, China). The recombinant plasmid was used to transform the competent *Escherichia coli* DH5α cells and sent to Invitrogen (Beijing, China) for sequencing. The resulting sequences were analyzed by a BLASTn search in GenBank and by using Clustal W method in the DNANAN V6 software (Lynnon Biosoft, San Ramon, CA, USA).

### Quantitative real-time PCR (qPCR)

Gene amplification was performed by qPCR in a Mx3005P qPCR system (Agilent Technologies, Santa Clara, USA) using TransStart^®^ Top Green qPCR SuperMix (TransGen Biotech) following the manufacturer’s instructions. The qPCR assays were conducted in 96-well polypropylene plates (Axygen, San Jose, USA) in a 20 μl reaction volume comprising 1 μl of cDNA template, 0.4 μl of each 10 μM primer (Table 1), 10 μl of 2× TransStart^®^ Top Green qPCR SuperMix, 0.4 μl of Passive Reference Dye (TransGen Biotech) and 7.8 μl of H_2_O. The thermal cycling program was as follows: 94 °C for 30 s, followed by 40 cycles of 94 °C for 5 s and 60 °C for 30 s. The primers with high amplification specificity were verified by different peaks observed in corresponding melting curves. Each plate contained triplicate reactions for each DNA sample. Melting curves were also traced after each assay to confirm that the fluorescence signal was retrieved from specific PCR products and to ensure the absence of primer dimers. The relative gene expression levels for each gene in each sample were calculated as Ct and transformed into relative values (RQ) by the 2^−ΔΔCT^ method, where ΔΔC_T_ = (C_T, Target_ − C_T, Actin_) _sample_ − (C_T, Target_ − C_T, Actin_) _control_ [30].

### Protein extraction

Eggs (1 g) were ground with a mortar and pestle in liquid nitrogen and dissolved in 20 mM sodium acetate buffer (pH 5.0). The homogenate was then centrifuged at 13,000× *rpm* for 10 min at 4 °C and the supernatant was transferred to a new tube for testing. The total protein concentration was determined according to Bradford [31] using bovine serum albumin (BSA) protein as the standard.

### Enzymatic assays

The activity of cathepsin B and D was assessed using Activity Fluorometric Assay Kits (BioVision, Milpitas, USA) following the manufacturer’s instructions. The Cathepsin B Activity Assay kit utilizes the preferred cathepsin B substrate sequence RR labeled with amino-4-trifluoromethyl coumarin (AFC). Samples that contain cathepsin B cleave the synthetic substrate RR-AFC to release free AFC. The released AFC can be quantified using a fluorometer or fluorescence plate reader at Ex/Em = 400/505 nm. The Cathepsin D Activity Assay kit utilizes the preferred cathepsin D substrate sequence GKPILFFRLK(Dnp)-D-R-NH2 labeled with 4-methylcoumarin-7-amide (MCA). Samples that contain cathepsin D cleave the synthetic substrate to release fluorescence, which can be quantified using a fluorometer or fluorescence plate reader at Ex/Em = 328/460 nm. The Acid Phosphatase Activity Assay kit uses p-nitrophenyl phosphate (pNPP) as a phosphatase substrate, which turns yellow (λmax = 405 nm) when dephosphorylated by acid phosphatase.

### Statistical analysis

Six biological replicates, representing different batches of separated spawning events, were performed for mRNA expression and enzymatic activity assessment. Data in all groups were analyzed using SPSS 12.0 software. The differences in mRNA expression and enzymatic activity among samples were compared by ANOVA. The differences were considered statistically significant when *P *< 0.05.

## Results

### Primer verification and qPCR conditions

**Fig. 1:**
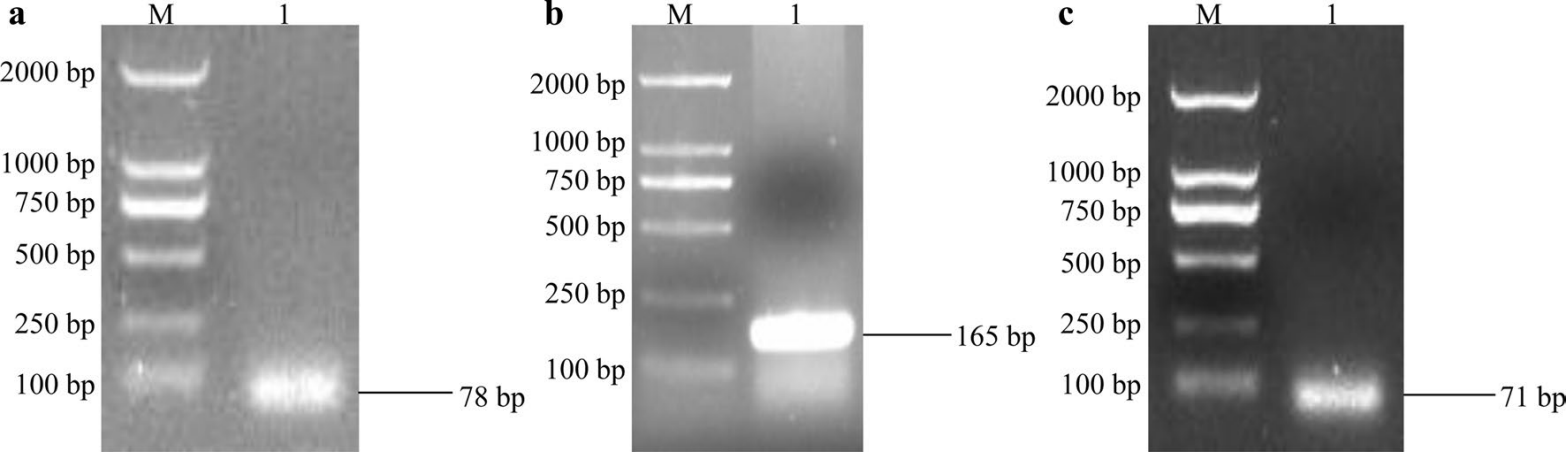
Primer verification by PCR amplification from cathepsin B (**a**), cathepsin D (**b**) and acid phosphatase (**c**) of *H. longicornis*

Non-specific amplification was performed to confirm each primer pair using the Tm shown in Table 1 with the cDNA template of target genes prepared before. Amplification products were analyzed by agarose gel electrophoresis where a single bright band was observed (Fig. 1a-c). PCR amplicons were sequenced to affirm the amplicon sequences for all genes evaluated in the present study.

### Cathepsin B dynamic changes during embryonic development

**Fig. 2:**
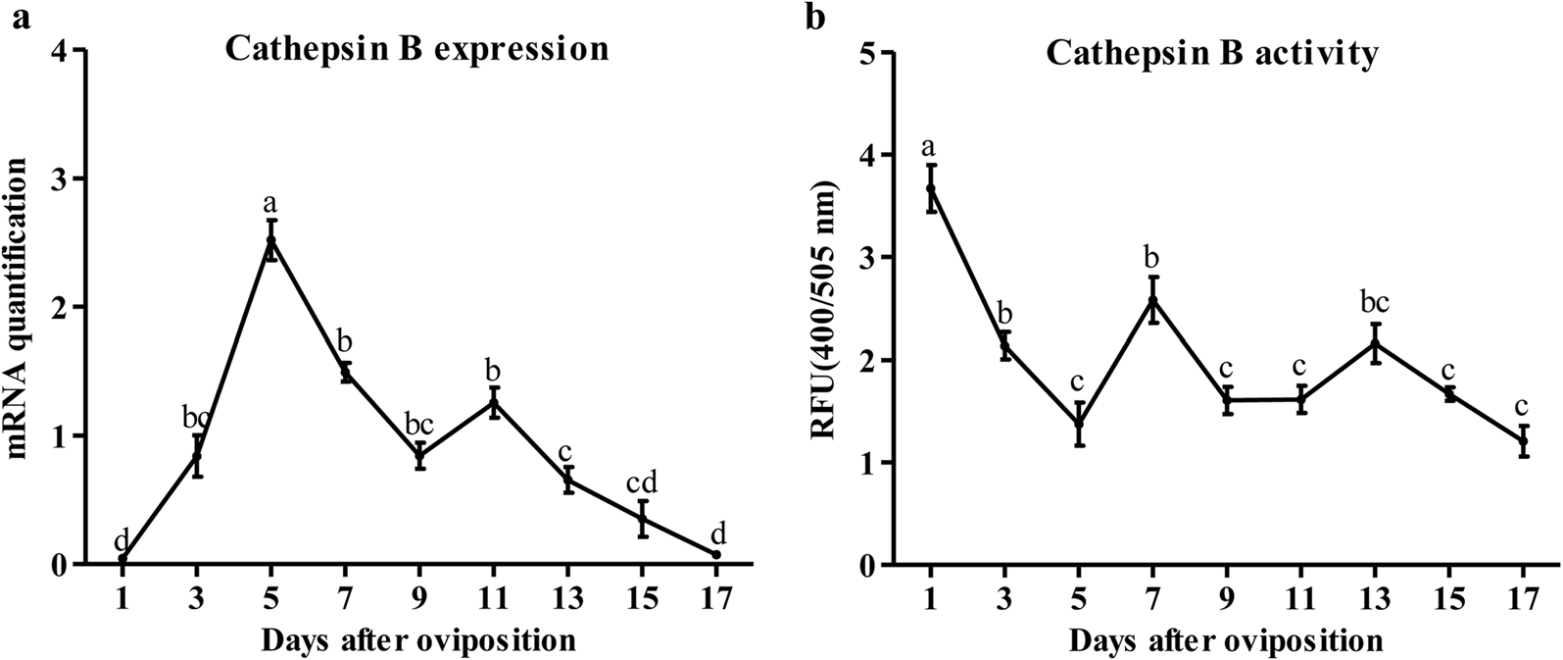
Expression (**a**) and enzymatic activity (**b**) profiling of cathepsin B during embryonic development in *H. longicornis*. Each point represents the mean ± SEM of three independent biological experiments and the different letters indicate significant differences (*P* < 0.05)

The relative expression levels of cathepsin B in the eggs from different stages of embryonic development were individually measured by quantitative real-time PCR. The results showed that cathepsin B was expressed almost throughout egg development except for the first and last day (Fig. 2a). An accumulation with significant upregulation of cathepsin B was observed in the eggs during the first five days and reached its highest expression on day 5 (ANOVA, *F*_(8,45)_ = 100.02, *P* < 0.0001). However, cathepsin B mRNA expression steadily decreased, along with the progress of embryonic development (Fig. 2a).

The enzymatic activity profile of cathepsin B is shown in Fig. 2b. A high activity level of cathepsin B was observed on the first day in comparison with later developmental stages. The activity decreased significantly on the third and fifth days and then remained relatively low until the end of egg development (ANOVA, *F*_(8,45)_ = 39.66, *P* < 0.0001) (Fig. 2b).

### Cathepsin D dynamic changes during embryonic development

**Fig. 3:**
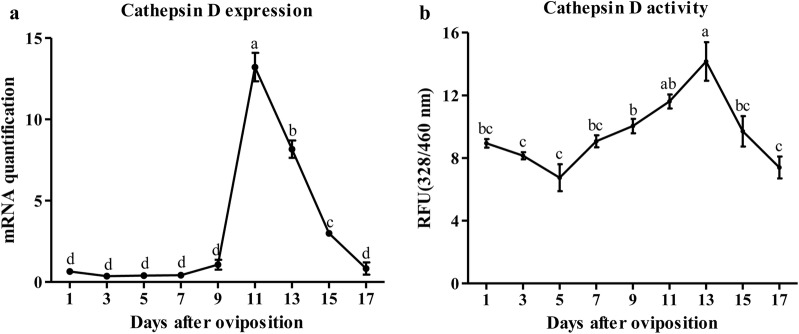
Expression (**a**) and enzymatic activity (**b**) profiling of cathepsin D during embryonic development in *H. longicornis*. Each point represents the mean ± SEM of three independent biological experiments and the different letters indicate significant differences (*P* < 0.05)

We analyzed cathepsin D expression profiles in different developmental stages of tick embryogenesis. At the transcriptional level, cathepsin D expression was deficient in the first nine days and obviously increased on day 11, but then rapidly and dramatically dropped (ANOVA, *F*_(8,45)_ = 269.59, *P* < 0.0001) (Fig. 3a).

As shown in Fig. 3b, cathepsin D activity was slowly reduced in the first three days but gradually increased during the following seven days. The highest activity of cathepsin D was observed on day 13 and then returned to the same level as in the first three days (ANOVA, *F*_(8,45)_ = 21.08, *P* < 0.0001) (Fig. 3b).

### Acid phosphatase dynamic changes during embryonic development

**Fig. 4:**
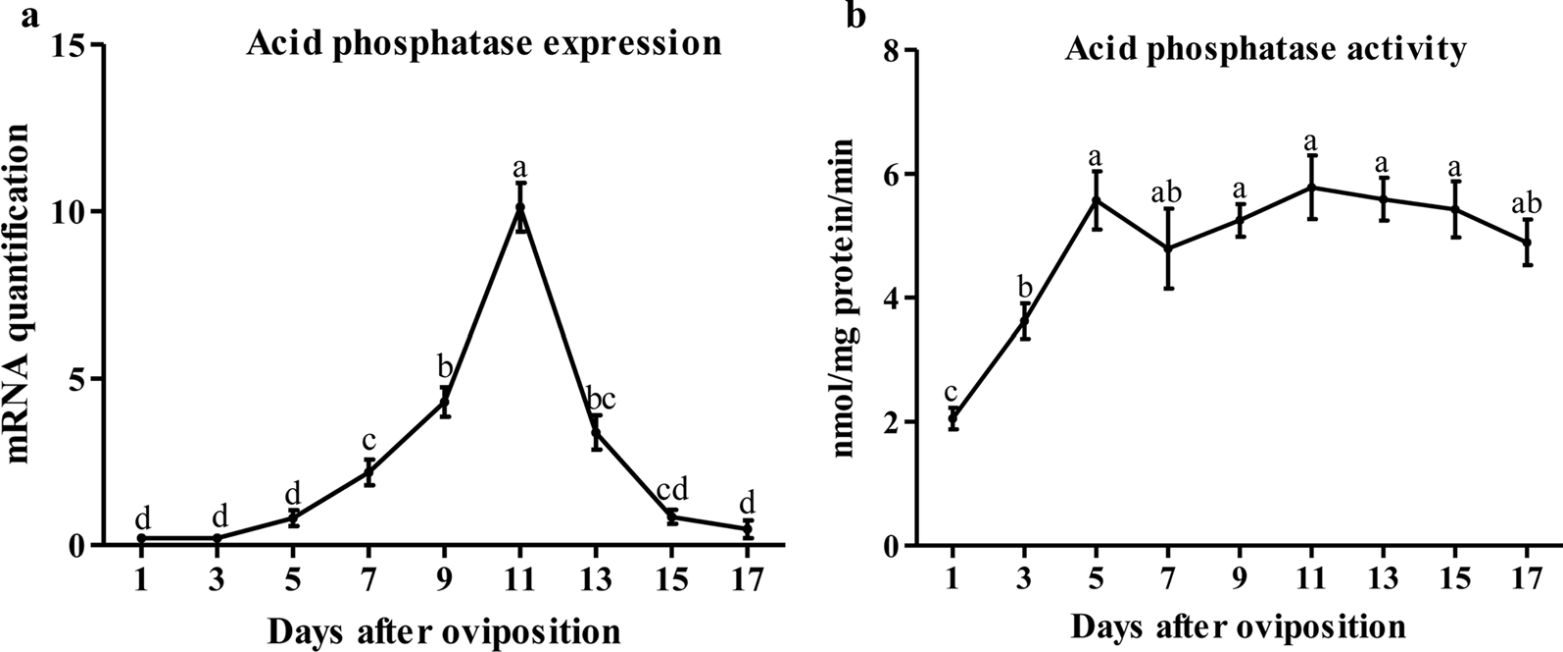
Expression (**a**) and enzymatic activity (**b**) profiling of acid phosphatase during embryonic development in *H. longicornis*. Each point represents the mean ± SEM of three independent biological experiments and the different letters indicate significant differences (*P* < 0.05)

For acid phosphatase, the relative expression analysis revealed that mRNA levels were first observed on day 5 and then accumulated with a significant increase (ANOVA, *F*_(8,45)_ = 140.08, *P* < 0.0001). It reached highest expression on day 11 and quickly reduced until the end of development (Fig. 4a).

Unlike cathepsin B and D, acid phosphatase activity increased significantly in the first five days, and then remained stable at high levels until the end of egg development (ANOVA, *F*_(8,45)_ = 17.05, *P* < 0.0001) (Fig. 4b).

## Discussion

Enzymes for protein hydrolysis in insect eggs are controlled scrupulously and orderly to ensure appropriate degradation of yolk proteins. These can be categorized into four types, namely cysteine proteases such as cathepsins B [9] and L [32], serine proteases [10], aspartic proteases [15] and acid phosphatases [14]. Among them, cathepsin B, cathepsin D and acid phosphatase have been frequently studied. In general, these enzymes are stored as an inactive form of a proenzyme in small yolk granules, but activated by the acidification of yolk granules, and then participate in digesting yolk proteins during embryonic development [33]. Analogous regulatory mechanisms in the degradation of yolk proteins are described in many species such as *Spodoptera exigua* [15], *Dipetalogaster maxima* [14], *Blatella germanica* [34], *R. prolixus* [16], *Bombyx mori* [8], *Musca domestica* [35] and *Culex quinquefasciatus* [9].

To evaluate the dynamic changes of cathepsin B, cathepsin D and acid phosphatase during Vn degradation and embryonic development in *H. longicornis*, we observed both mRNA expression and activity of these three enzymes during egg development from a very early stage until hatching. The results showed that all three enzymes were expressed during egg development, which agrees with that observed in insects in other studies [11, 16, 19]. Both cathepsin D and acid phosphatase showed the highest mRNA expression on day 11, and cathepsin B on day 5. All three enzymes showed high activity in the eggs, but the activity patterns were not consistent with the expression profiles. The accumulation of three enzyme transcripts levels and high activity in the eggs may suggest their participation in the proteolytic process, where Vn is partially cleaved into small peptides and free amino acids [33].

Cathepsin B is a kind of proteinase in cytolysosome, which plays a crucial role in yolk utilization in oviparous animals and is able to cleave peptide bonds of their substrates [12]. In this study, cathepsin B mRNA expression significantly increased during early development (1–5 days) in the eggs but immediately decreased after that. Two noticeable decreases were observed on days 7 and 13, respectively. This expression profile is different from others such as *Helicoverpa armigera* and *Seriola lalandi*, in which cathepsin B showed the highest expression on the first day and gradually decreased during embryonic development [11, 36]. The variation tendency was, however, consistent with *M. domestica* [37]. The expression profile of cathepsin B is not uniform across different species. It is reported that increased expression of cathepsin B occurred throughout development in *Fundulus heteroclitus* [38], whereas in *Phenacoccus solenopsis*, the expression level was relatively low at the egg stage [39]. Nevertheless, the activity pattern of cathepsin B in *H. longicornis* is consistent with the results in *He. armigera*, which showed a steady decline during egg development [11].

Cathepsin D belongs to lysosomal aspartic protease, which is nearly ubiquitously expressed. This enzyme participates in several physiological processes such as protein degradation, autophagy, programmed apoptosis regulation and enzymatic precursor activation [40]. The mRNA expression and enzymatic activity of cathepsin D have been well analyzed in many insect species and a recent study showed that cathepsin D might function during metamorphosis and represent a target for insect control [15]. In this study, cathepsin D expression was practically undetectable from day 0 to day 9, began to rise straightly on day 11, and then reduced significantly until the end of development. Consistent with this result, the trend of cathepsin D expression in *B. mori* is also first increased and then subsequently decreased [41]. The activity of this enzyme gradually accumulated during the first 13 days and significantly reduced at the end of development. This finding is similar to the activity patterns during embryogenesis in *R. prolixus* [16].

Acid phosphatases are known as hydrolases which are often associated with degradation of proteins. They usually described as lysosomal markers [14]. Our results revealed that the transcripts of acid phosphatase were extremely low during the first three days and the following trend was similar to cathepsin D, showing the highest expression on day 11. The acid phosphatase activity gradually increased from day 0 to 5, reaching a peak on day 5, and remained active until the end of egg development. This result was similar to studies in *P. americana* [19] and *R. microplus* [21], in which activity reached a peak on day 6 and 17, respectively.

The reproduction and development of ticks highly rely on Vn degradation during embryogenesis. Various proteolytic enzymes are responsible for utilization of yolk proteins during embryonic development in hematophagous arthropods and insects, including acidic cysteine proteases such as cathepsins B [9] and L [32], serine proteases [10], aspartic proteases [15] and also acid phosphatases [14]. During egg development, yolk granules are acidized, which is able to trigger the activation of proenzymes and yolk proteolysis [20]. This process has been proved in many models such as cysteine proteases in *B. germanica* and *B. mori* [34, 42], which can be activated by the acidification at embryogenesis. It has been indicated that in *P. americana*, pH acidification might be a possible mechanism for proenzymes activation during embryogenesis [19]. Moreover, acidification of yolk granules also does not occur homogenously in invertebrate eggs. In the starfish *Pisaster ochraceus*, researchers detected differences of egg proteins in cortical granule populations [43]. Our results showed that the expression patterns of the three enzymes were different from their activity. Therefore, we think the activity of these enzymes may be controlled by the populations of acidifying yolk granules and egg proteins, which may also supervise the limited proteolysis of Vn during embryogenesis. This phenomenon was also found in ovarian tissue of *D. maxima*, where both pro-cathepsin D and cathepsin D were detected at all reproductive stages, but the highest cathepsin D activity was found at early and late atresia [14]. It is noteworthy that cathepsin B showed the highest expression on day 5, and cathepsin D as well as acid phosphatase showed the highest expression on day 11. According to Friesen et al. [44], embryos of *Dermacentor andersoni* completed an extension of germ band around yolk on day 5, while on day 11, the differentiation of leg segments became clear and palp endites have begun to fuse. As such, we speculate that in addition to the roles in these two stages of Vn degradation, these three enzymes also participate in tissue differentiation and organ formation during embryonic development, which results in the difference between mRNA expression and enzymatic activity.

In arthropods, the interaction effect between acid phosphatase and other hydrolases on Vn degradation during embryogenesis has been mentioned. In *D. maxima*, *R. microplus* and *R. prolixus*, acid phosphatase has been proved to cooperate with aspartyl protease to degrade Vn during embryogenesis [14, 16, 21]. Furthermore, the correlation between acid phosphatase and cysteine peptidase on Vn degradation has been demonstrated in *P. americana* [19]. Conversely, although acid phosphatase and cathepsin B in *M. domestica* showed similar activity patterns during embryogenesis, the inhibition of acid phosphatase still did not affect yolk protein hydrolysis by cathepsin [37]. Unfortunately, the association among acid hydrolases is still unclear, and evidence for these processes has not yet been found in most species of ticks. In future studies, we should investigate the relationship and interaction among the three enzymes, and clarify their effect on Vn degradation and embryogenesis.

## Conclusions

In this study, we investigated the mRNA expression and enzymatic activity of cathepsins B, D and acid phosphatase during embryonic development in *H. longicornis*. All three enzymes were not only expressed and activated in eggs but also presented dynamic changes with the development of embryos. Their transcription levels and activity profiles demonstrated for the first time in *H. longicornis*, that the three enzymes were existent and had different change patterns during egg development, indicating they were orderly controlled and played various roles in Vn degradation. However, further studies are required to explore the functions of these enzymes in Vn degradation during embryogenesis and their interaction. This will contribute to selecting the best potential molecular target and figuring out protective antigen for controlling tick life-cycle and disease transmission.

## Abbreviations

Vn: vitellin
RH: relative humidity
L:D: light:dark photocycle
PCR: polymerase chain reaction
qPCR: quantitative real-time PCR
RQ: relative quantity
BSA: bovine serum albumin
AFC: amino-4-trifluoromethyl coumarin
MCA: 4-methylcoumarin-7-amide
pNPP: p-nitrophenyl phosphate
Tm: melting temperature

## References

[1] Chen Z, Yang XJ, Bu FJ, Yang XH, Yang XL, Liu JZ (2010). Ticks (Acari: Ixodoidea: Argasidae, Ixodidae) of China. Exp Appl Acarol.

[2] Yu ZJ, Wang H, Wang TH, Sun WY, Yang XL, Liu JZ (2015). Tick-borne pathogens and the vector potential of ticks in China. Parasit Vectors..

[3] De la Fuente J, Contreras M, Estradapeña A, Cabezascruz A (2017). Targeting a global health problem: vaccine design and challenges for the control of tick-borne diseases. Vaccine..

[4] Wang RR, Li NX, Liu JN, Li T, Liu M, Yu ZJ (2017). Symbiont dynamics of the Tibetan tick *Haemaphysalis tibetensis* (Acari: Ixodidae). Parasit Vectors..

[5] Liu JZ, Yang XJ (2013). Ticks.

[6] Umemiya-Shirafuji R, Tanaka T, Boldbaatar D, Tanaka T, Fujisaki K (2012). Akt is an essential player in regulating cell/organ growth at the adult stage in the hard tick *Haemaphysalis longicornis*. Insect Biochem Mol Biol.

[7] Estrela AB, Seixas A, Teixeira VO, Pinto AF, Termignoni C (2010). Vitellin-and hemoglobin-digesting enzymes in *Rhipicephalus* (*Boophilus*) *microplus* larvae and females. Comp Biochem Phys B..

[8] Cai XY, Yu J, Yu HY, Liu YW, Fang Y, Ren ZX (2014). Core promoter regulates the expression of cathepsin B gene in the fat body of *Bombyx mori*. Gene.

[9] Moura AS, Cardoso AF, Costa da Silva AL, Winter CE, Bijovsky AT (2015). Two cathepsins B are responsible for the yolk protein hydrolysis in *Culex quinquefasciatus*. PLoS One..

[10] Wang DD, Yan Z, Dong ZM, Guo PC, Ma SY, Guo KY (2016). Serine protease P-IIc is responsible for the digestion of yolk proteins at the late stage of silkworm embryogenesis. Insect Biochem Mol Biol.

[11] Zhao XF, An XM, Wang JX, Dong DJ, Du XJ, Sueda S (2005). Expression of the *Helicoverpa* Cathepsin B-like proteinase during embryonic development. Arch Insect Biochem Phys..

[12] Pezhman M, Hosseini SM, Ostadhosseini S, Varnosfaderani SR, Sefid F, Nasr-Esfahani MH (2017). Cathepsin B inhibitor improves developmental competency and cryo-tolerance of *in vitro* ovine embryos. BMC Dev Biol.

[13] Pohl PC, Sorgine MH, Leal AT, Logullo C, Oliveira PL, Vaz Ida S (2008). An extraovarian aspartic protease accumulated in tick oocytes with vitellin-degradation activity. Comp Biochem Phys B..

[14] Leyria J, Fruttero LL, Nazar M, Canavoso LE (2015). The role of DmCatD, a cathepsin D-like peptidase, and acid phosphatase in the process of follicular atresia in *Dipetalogaster maxima* (Hemiptera: Reduviidae), a vector of Chagasʼ disease. PLoS One..

[15] Kang T, Jin R, Zhang Y, Wang H, Lee KS, Jin BR (2017). Functional characterization of the aspartic proteinase cathepsin D in the beet armyworm (*Spodoptera exigua*). Gene.

[16] Fialho E, Nakamura A, Juliano L, Masuda H, Silvaneto MA (2005). Cathepsin D-mediated yolk protein degradation is blocked by acid phosphatase inhibitors. Arch Biochem Biophys.

[17] Fuzita FJ, Pinkse MW, Verhaert PD, Lopes AR (2015). Cysteine cathepsins as digestive enzymes in the spider *Nephilengys cruentata*. Insect Biochem Mol Biol.

[18] Zhang P, Tian ZC, Liu GY, Xie JR, Luo J, Zhang LY (2011). Characterization of acid phosphatase from the tick *Haemaphysalis longicornis*. Vet Parasitol.

[19] Oliveira DM, Ramos IB, Reis FC, Lima AP, Machado EA (2008). Interplay between acid phosphatase and cysteine proteases in mediating vitellin degradation during early embryogenesis of *Periplaneta americana*. J Insect Phys..

[20] Abreu LA, Valle D, Manso PP, Façanha AR, Pelajo-Machado M, Masuda H (2004). Proteolytic activity of *Boophilus microplus* Yolk pro-Cathepsin D (BYC) is coincident with cortical acidification during embryogenesis. Insect Biochem Mol Biol.

[21] Silveira AB, Castro-Santos J, Senna R, Logullo C, Fialho E (2006). Tick vitellin is dephosphorylated by a protein tyrosine phosphatase during egg development: effect of dephosphorylation on VT proteolysis. Insect Biochem Mol Biol.

[22] Nascimento-Silva MC, Leal AT, Daffre S, Juliano L, da Silva Vaz IJ, Paiva-Silva GO (2008). BYC, an atypical aspartic endopeptidase from *Rhipicephalus* (*Boophilus*) *microplus* eggs. Comp Biochem Phys B..

[23] Oldiges DP, Parizi LF, Zimmer KR, Lorenzini DM, Seixas A, Masuda A (2012). A *Rhipicephalus* (*Boophilus*) *microplus* cathepsin with dual peptidase and antimicrobial activity. Int J Parasitol.

[24] Zheng HY, Yu ZJ, Zhou LF, Yang XL, Liu JZ (2012). Seasonal abundance and activity of the hard tick *Haemaphysalis longicornis* (Acari: Ixodidae) in North China. Exp Appl Acarol.

[25] Lee DW, Chang KS, Min JK, Ahn YJ, Jo HC, Kim SI (2015). Acaricidal activity of commercialized insecticides against *Haemaphysalis longicornis* (Acari: Ixodidae) nymphs. J Asia Pac Entomol..

[26] Rainey T, Occi JL, Robbins RG, Egizi A (2018). Discovery of *Haemaphysalis longicornis* (Ixodida: Ixodidae) parasitizing a sheep in New Jersey, United States. J Med Entomol..

[27] Yang XL, Yu ZJ, Gao ZH, Yang XH, Liu JZ (2014). Morphological characteristics and developmental changes of the ovary in the tick *Haemaphysalis longicornis* Neumann (Acari: Ixodidae). Med Vet Entomol.

[28] Yang XL, Yu ZJ, He YJ, Xu XL, Gao ZH, Wang H (2015). Purification of vitellin and dynamics of vitellogenesis in the parthenogenetic *Haemaphysalis longicornis* (Acari: Ixodidae). Exp Appl Acarol.

[29] Liu J, Liu Z, Zhang Y, Yang X, Gao Z (2005). Biology of *Dermacentor silvarum* (Acari: Ixodidae) under laboratory conditions. Exp Appl Acarol.

[30] Livak KJ, Schmittgen TD (2001). Analysis of relative gene expression data using real-time quantitative PCR and the 2^−ΔΔCT^ Method. Methods.

[31] Bradford MM (1976). A rapid and sensitive method for the quantitation of microgram quantities of protein utilizing the principle of protein-dye binding. Anal Biochem.

[32] Fagotto F (1990). Yolk degradation in tick eggs: 1. Occurrence of a cathepsin L-like acid proteinase in yolk spheres. Arch Insect Biochem..

[33] Giorgi F, Bradley JT, Nordin JH (1999). Differential vitellin polypeptide processing in insect embryos. Micron..

[34] Liu X, McCarron RC, Nordin JH (1996). A cysteine protease that processes insect vitellin. Purification and partial characterization of the enzyme and the proenzyme. J Biol Chem..

[35] Padilha MH, Pimentel AC, Ribeiro AF, Terra WR (2009). Sequence and function of lysosomal and digestive cathepsin D-like proteinases of *Musca domestica* midgut. Insect Biochem Mol Biol.

[36] Palomino J, Herrera G, Torres-Fuentes J, Dettleff P, Patel A, Martínez V (2017). Assessment of cathepsin mRNA expression and enzymatic activity during early embryonic development in the yellowtail kingfish *Seriola lalandi*. Anim Reprod Sci..

[37] Ribolla PEM, Daffre S, Bianchi AGD (1993). Cathepsin B and acid phosphatase activities during *Musca domestica*, embryogenesis. Insect Biochem Mol Biol.

[38] Tingaud-Sequeira A, Carnevali O, Cerdà J (2011). Cathepsin B differential expression and enzyme processing and activity during *Fundulus heteroclitus* embryogenesis. Comp Biochem Phys A..

[39] Luo M, Dong ZY, Shuying B, Liao HZ, Lin JT (2012). Molecular cloning, prokaryotic expression and expression at different developmental stages of cathepsin B gene in mealybug *Phenacoccus solenopsis* Tinsley (Hemiptera: Pseudococcidae). Acta Entomol Sin..

[40] Mehanna S, Suzuki C, Shibata M, Sunabori T, Imanaka T, Araki K (2015). Cathepsin D in pancreatic acinar cells is implicated in cathepsin B and L degradation, but not in autophagic activity. Biochem Biophys Res Commun.

[41] Gui ZZ, Lee KS, Kim BY, Choi YS, Wei YD, Choo YM (2006). Functional role of aspartic proteinase cathepsin D in insect metamorphosis. BMC Dev Biol.

[42] Yamahama Y, Uto N, Tamotsu S, Miyata T, Yamamoto Y, Watabe S (2003). *In vivo* activation of pro-form *Bombyx* cysteine protease (BCP) silk moth eggs: localization of yolk proteins and BCP, and acidification of yolk granules. J Insect Physiol.

[43] Gerhartz B, Auerswald EA, Mentele R, Fritz H, Macheidt W, Kolb HJ (1997). Proteolytic enzymes in yolk-sac membrane of quail egg. Purification and enzymatic characterization. Comp Biochem Physiol..

[44] Friesen KJ, Dixon M, Lysyk TJ (2015). Embryo development and morphology of the rocky mountain wood tick (Acari: Ixodidae). J Med Entomol.

